# Comparison of Long-Term Survival Between cT1N0 Stage Esophageal Cancer Patients Receiving Endoscopic Dissection and Esophagectomy: A Meta-Analysis

**DOI:** 10.3389/fsurg.2022.917689

**Published:** 2022-05-06

**Authors:** Wei Lu, Peng Li, Wu Wen, Yi Jian

**Affiliations:** Department of Gastroenterology, Chengdu Second People’s Hospital, Chengdu, Sichuan, China

**Keywords:** endoscopic dissection, esophagectomy, cT1N0, esophageal carcinoma, meta-analysis

## Abstract

**Background:**

Endoscopic dissection (ED) shows relatively high clinical value in early esophageal cancer (cT1N0) such as lower incidence of postoperative complications and hospitalization costs and enhanced recovery. However, whether ED still has certain advantages over esophagectomy in terms of long-term survival remains unclear.

**Purpose:**

The aim of this meta-analysis was to compare the long-term outcomes of ED and surgery in the treatment of cT1N0 esophageal cancer.

**Methods:**

Several electronic databases including the PubMed, EMBASE, Web of Science and Cochrane Library databases were searched up to April 7, 2022 for studies which compared the overall survival (OS) and disease-specific survival (DSS) of cT1N0 esophageal cancer patients receiving the ED or esophagectomy. The hazard ratios (HRs) and 95% confidence intervals (CIs) were combined and all statistical analysis was conducted through STATA 15.0 software.

**Results:**

A total of 12 studies involving 3,732 patients were enrolled. No significant difference in the OS between ED and surgery groups was observed (HR = 0.78, 95% CI, 0.59–1.04, *p* = 0.089). However, the DSS of the ED group was significantly longer than that of the surgery group (HR = 0.56, 95% CI, 0.39–0.82, *p* = 0.003).

**Conclusion:**

In overall, the current evidence manifested that the long-term survival of cT1N0 esophageal cancer patients undergoing ED was not worse than that of patients undergoing esophagectomy. ED may be considered as the primary treatment for cT1N0 esophageal carcinoma patients.

## Introduction

Esophageal cancer is one of the most common tumors with high mortality rate worldwide (1–3). Esophagectomy remains the primary treatment option for most cases (4, 5). However, the incidence of surgery-related complications is still relatively high and patient’s quality of life declines significantly postoperatively despite of the great advances in the surgical techniques in recent years (6, 7). With the improvement of the general public’s health awareness, the early screening of esophageal carcinoma is becoming more and more popular (8, 9). As a result, the proportion of superficial esophageal carcinoma which is confined to the mucosa among all esophageal cancer cases is also continuing increasing (10, 11). In addition to curing the disease, patients are increasingly demanding in terms of therapeutic risk, cost and quality of life.

Esophagoscope was originally designed to examine esophageal lesions. However, its clinical value in the treatment of superficial esophageal disease is gradually manifested. Besides, previous literatures have demonstrated that endoscopic dissection (ED) is obviously related to lower risk of adverse events and procedure-related mortality rate in early stage esophageal cancer patients (12). Furthermore, ED has significant advantages in terms of economic benefits over the surgery (13, 14). Thus, ED is believed to show high clinical value in treatment early stage (T1N0) esophageal cancer. However, due to the highly malignant and aggressive nature of esopahgeal cancer, long-term prognosis of patients receiving ED should be of great concern. Whether ED still has certain advantages in terms of long-term survival is unclear in cT1N0 esophageal cancer patients.

Therefore, the aim of this meta-analysis was to compare the long-term outcomes of ED and esophagectomy for cT1N0 esophageal cancer patients, which might contribute to the clinical treatment and management for this group of patients.

## Materials and Methods

This meta-analysis was conducted according to the Preferred Reporting Items for Systematic Reviews and Meta-Analyses guidelines (15).

### Literature Search

The PubMed, EMBASE, Web of Science and Cochrane Library databases electronic databases were searched up to April 7, 2022 for relevant studies. The following terms were used during the literature search: endoscopic submucosal dissection, endoscopic mucosal dissection, esophagus, esophageal, tumor, cancer, carcinoma, neoplasm and esophagectomy. In detail, the specific search strategy was as follows: (endoscopic submucosal dissection OR endoscopic mucosal dissection) AND (esophagus OR esophageal) AND (tumor OR cancer OR neoplasm OR carcinoma) AND esophagectomy. Besides, the references cited in included papers were also reviewed for availability.

### Inclusion and Exclusion Criteria

The inclusion criteria were as follows: (1) patients were diagnosed with primary esophageal cancer; (2) the clinical stage was with cT1N0 which indicated that tumor confined to mucosa or submucosa; (3) patients received the ED including the endoscopic mucosal dissection (EMD) and endoscopic submucosal dissection (ESD) or esophagectomy and patients receiving the ED and then esophagectomy were divided into the surgery group; (4) the long-term survival representing as overall survival (OS) and disease-specific survival (DSS) including the disease-free survival (DFS), recurrence-free survival (RFS) and cancer-specific survival (CSS) was compared between ED and surgery groups; (5) HRs with 95% CIs were reported or enough data were provided to calculate them.

The exclusion criteria were as follows: (1) duplicated or overlapped data; (2) other anti-tumor interventions were involved during the comparison between ED and esophagectomy; (3) meeting abstracts, editorials, letters, reviews or case reports; (4) low quality studies with the Newcastle Ottawa Scale (NOS) score of 5 or lower (16).

### Data Extraction

The following data were collected form each included studies: the name of author, publication year, country, sample size, number of patients receiving ED, stage (T1a or T1b), tumor type, endpoint and hazard ratios (HRs) with corresponding 95% confidence intervals (CIs).

### Methodological Quality Assessment

The quality of included studies were evaluated according to the NOS, and studies with a NOS score of 6 or higher were defined as high-quality studies and could be included (16).

The literature search, selection, data extraction and methodological quality evaluation were all performed by two authors independently. Any disagreement was resolved by team discussion.

### Statistical Analysis

All statistical analysis was conducted by STATA 15.0 software. The HR with 95%CI were combined to assess the association between the treatment method and long-term survival of cT1N0 stage esophageal cancer patients. If the HRs with 95% CIs were not reported directly, then they would be calculated from Kaplan-Meier curves with the method described by Tierney et al. (17). The heterogeneity was evaluated by Cochran’s Q test and Higgins *I*^2^ statistic; *P*_heterogeneity_ < 0.10 and/or *I*^2^ > 50% was defined as significant heterogeneity among studies, and the random-effects model was applied for the pooled effect estimates, otherwise the fixed-effects model was used (18). Subgroup analyses stratified by the tumor stage (T1a vs. T1) and tumor type were further conducted. Begg’s funnel plot and Egger’s test were conducted to evaluate publication bias and significant publication bias was defined as *p* < 0.05 (19).

## Results

### Literature Search and Selection

**Figure 1:**
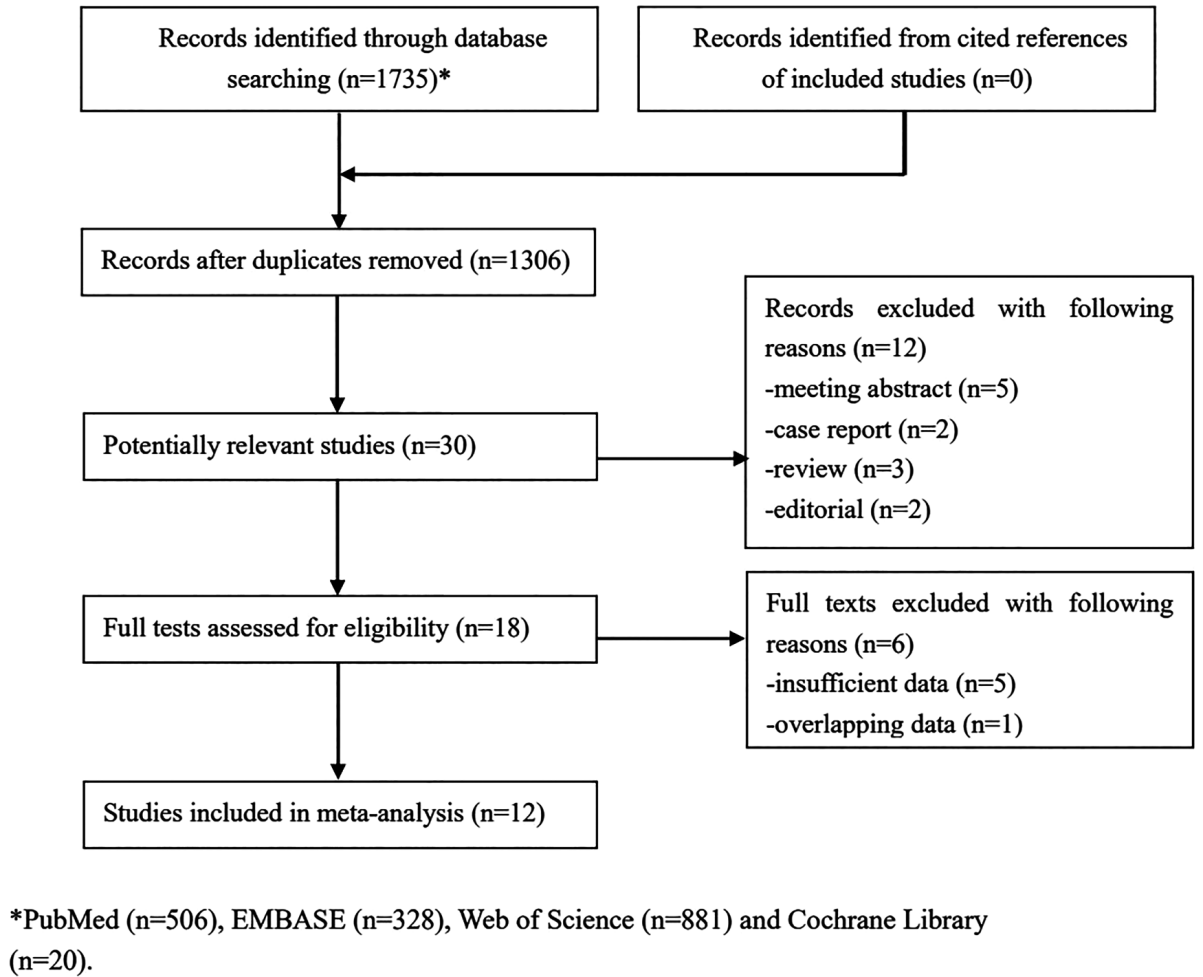
The flow diagram of this meta-analysis.

Initially, 1,735 records were searched from databases and 429 duplicated records were removed. Then after scanning the titles, 1,276 irrelevant publications were excluded. After reviewing the abstracts of 30 potentially related publications, 18 studies were then excluded. Finally, a total of 12 studies were included in this meta-analysis (20–31). The detailed selection process was presented in Figure 1.

### Basic Characteristics of Included Studies

Among the 12 studies, 3,732 patients were involved (20–31) with the sample size ranged from 100 to 954 and 1,671 patients received ED. Three studies focused on T1a stage patients whose tumor was confined to the mucosa (21, 22, 30). Besides, six and three studies focused on squamous cell carcinoma (SCC) (23, 25–28, 30) and adenocarcinoma (AC) (20–22, 29) cases. All studies were regarded as high-quality studies with the NOS score of 6 or higher. The other detailed information was shown in Table 1.

**Table 1:** Basic characteristics of included studies.

Author	Year	Country	Sample size	Number of ED	Stage	Tumor type	Endpoint	Source of HR	NOS
Pech (20)	2011	Germany	114	76	T1	AC	OS	E	7
Zehetner (21)	2011	USA	101	40	T1a	AC	OS	E	7
Schmidt (22)	2016	USA	100	36	T1a	AC	OS	E	7
Min (23)	2018	Korea	240	120	T1	SCC	OS, DSS	R	7
Qin (24)	2019	USA	954	224	T1	Mixed	OS, DSS	R	8
Yuan (25)	2019	China	116	69	T1	SCC	OS	E	7
Zhang (26)	2019	China	596	322	T1	SCC	OS, DSS	R/E	7
An (27)	2020	China	406	222	T1	SCC	OS	R	8
Lee (28)	2020	Republic Korea	184	70	T1	SCC	OS, DSS	R	8
Saunders (29)	2020	UK	189	93	T1	AC	DSS	R	7
Kim (30)	2021	Korea	463	263	T1a	SCC	OS	R	7
Dunn (31)	2022	UK	269	136	T1	Mixed	OS, DSS	R	8

*ESD, endoscopic dissection; HR, hazard ratio; NOS, Newcastle-Ottawa Scale; AC, adenocarcinoma; SCC, squamous cell carcinoma; OS, overall survival; DSS, disease-specific survival; E, estimated; R, reported.*

### Comparison of OS between Patients Receiving ED and Esophagectomy

**Figure 2:**
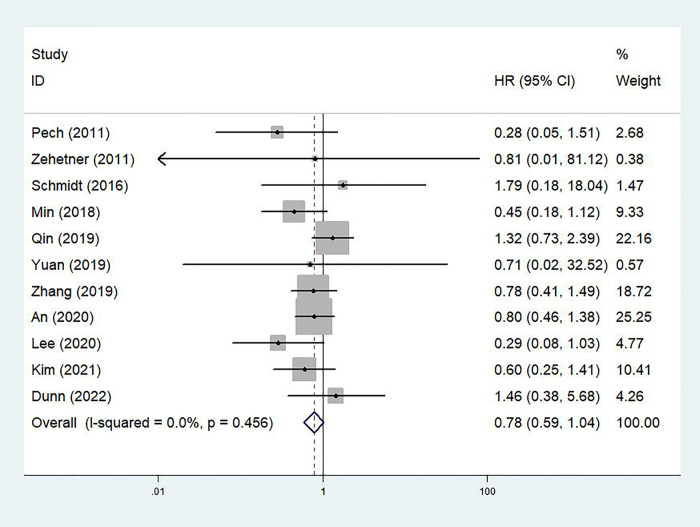
Comparison of overall survival between patients receiving endoscopic dissection and esophagectomy.

A total of 11 studies explored the association between the treatment option and OS of cT1N0 stage esophageal cancer patients (20–28, 30, 31). The pooled results indicated that no significant difference between the ED and surgery groups were observed (HR = 0.78, 95% CI: 0.59–1.04, *p* = 0.089; *I*^2 ^= 0.0%, *P*_heterogeneity_ = 0.456) (Figure 2). Subgroup analysis based on the stage showed similar results. However, subgroup analysis stratified by the tumor type manifested that ED was superior to esophagectomy in terms of OS for cT1N0 stage esophageal SCC patients (HR = 0.65, 95% CI, 0.47–0.91, *p* = 0.013; *I*^2 ^= 0.0%, *P*_heterogeneity_ = 0.696) (Table 2).

**Table 2:** Results of meta-analysis.

	No. of studies	HR	95% CI	*p* value	*I*^2^ (%)	*P*_heterogeneity_
Overall survival	11	0.78	0.59–1.04	0.089	0.0	0.456
Stage						
T1	8	0.80	0.59–1.08	0.140	21.7	0.257
T1a	3	0.69	0.31–1.53	0.362	0.0	0.683
Tumor type						
Adenocarcinoma	3	0.56	0.15–2.07	0.384	0.0	0.441
Squamous cell carcinoma	6	0.65	0.47–0.91	**0**.**013**	0.0	0.696
Disease-specific survival	6	0.56	0.39–0.82	**0**.**003**	19.5	0.287
Tumor-type				** **		
Squamous cell carcinoma	3	0.60	0.35–1.02	0.057	0.0	0.872
Adenocarcinoma	1	0.29	0.14–0.61	**0**.**001**	-	-

*HR, hazard ratio; CI, confidence interval.* *Bold values indicate significant statistical difference.*

### Comparison of DSS between Patients Receiving ED and Esophagectomy

**Figure 3:**
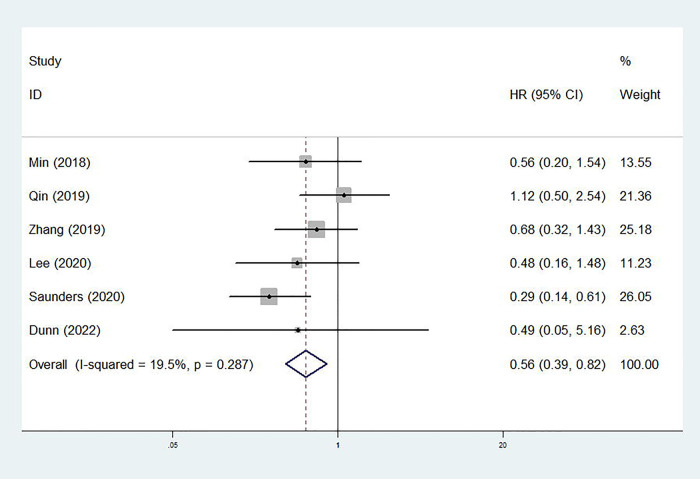
Comparison of disease-specific survival between patients receiving endoscopic dissection and esophagectomy.

Only six studies explored the association between the treatment option and DSS of cT1N0 stage esophageal cancer patients (23, 24, 26, 28, 29, 31). The pooled results demonstrated that the DSS of patients in the ED group was significantly longer than that in the surgery group (HR = 0.56, 95% CI, 0.39–0.82, *p* = 0.003; *I*^2 ^= 19.5%, *P*_heterogeneity_ = 0.287) (Figure 3). For AC patients, similar results were observed (HR = 0.29, 95% CI, 0.14–0.61, *p* = 0.001). However, no significant difference between the ED and surgery groups in SCC patients was observed (HR = 0.60, 95% CI, 0.35–1.02, *p* = 0.057; *I*^2 ^= 0.0%, *P*_heterogeneity_ = 0.872) (Table 2).

### Publication Bias

**Figure 4:**
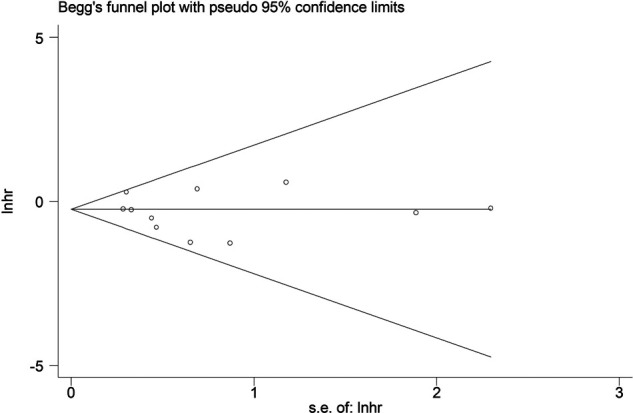
Begg’s funnel plot.

Due to the symmetric Begg’s funnel plot (Figure 4) and *p* = 0.483 for Egger’s test, no significant publication bias was detected in this meta-analysis.

## Discussion

The current meta-analysis demonstrated that the long-term prognosis of cT1N0 esophageal cancer patients undergoing ED was not worse than that of patients undergoing esophagectomy after including 12 studies and 3,732 patients. Meanwhile, ED was even superior to esophagectomy in some cases. Thus, based on the evidence provided by our study and previous literatures, ED could be considered as the primary treatment for cT1N0 stage esophageal cancer. However, more prospective high quality clinical trials are still needed to verify above findings.

Actually, in addition to the long-term survival, a number of studies have explored the clinical role of ED in terms of other fields. In the meta-analysis conducted by Zheng et al., 2,467 patients undergoing ED and 2,264 patients undergoing surgery were enrolled (12). Their results demonstrated that patients receiving ED showed significantly lower incidence of major adverse events [relative risk (RR) = 0.46, 95% CI, 0.33–0.64, *p* < 0.001] and procedure-related mortality (RR = 0.27, 95% CI, 0.10–0.73, *p* < 0.001) than those receiving esophagectomy (12). Besides, several researches compared the hospitalization cost of patients between the ED and surgery groups and their results manifested that the hospitalization costs in the surgery group was significantly higher than that in the ED group (13, 14, 25, 26). Meanwhile, the length of stage in the ED group was also obviously shorter than that in the esophagectomy group (13, 14, 25, 26, 32). Considering the above mentioned aspects, ED is obviously better than esophagectomy in treatment of early stage esophageal cancer.

However, some scholars suggested that ED might show a lower R0 resection rate due to the operational limitations (13, 20, 22, 25, 26, 32). Thus, patients undergoing ED may be at higher risk of relapse compared to patients undergoing esophagectomy. After comprehensively reviewing previous relevant literatures focusing on T1N0 stage esophageal cancer patients, the recurrence rates in the esophagectomy group and ED group ranged from 0% to 28.6% and from 1.79% to 13.0%. In overall, there was no obvious difference in the recurrence rate between the two groups [odds ratio (OR) = 1.24, 95% CI, 0.73–2.10, *p* = 0.420] (20–22, 25, 26, 32–39). Identifying the long-term efficacy of ED for superficial esophageal cancer has been an issue we need to address. Therefore, we designed the current meta-analysis and demonstrated that ED remained non-inferior to esophagectomy in terms of long-term survival for cT1N0 stage esophageal carcinoma patients.

Actually, we deem that there are still some valuable fields worthy of more depth investigations about the ED for early stage esophageal cancer. For example, some scholars indicated that T1b stage patients who undergoing ESD should receive adjuvant therapies like the chemotherapy, but others had different opinions (40). Furthermore, it is also necessary to compare the therapeutic effects of ED combined chemotherapy and esophagectomy for cT1N0 stage esophageal cancer patients. Besides, our meta-analysis revealed that for specific population ED might show higher prognostic value than esophagectomy, which should be verified by more high-quality studies with big sample sizes. The operative skills of endoscopists might be closely related to the therapeutic effects of ED and future studies should take this into account.

There are several limitations in this meta-analysis. First, all included studies are retrospective with relatively small sample sizes, which might cause some bias. Second, stage is believed to be an essential factor affecting the treatment option. However, we were unable to conduct more detailed subgroup stratified by the stage (T1a vs. T1b) due to the lack of original data and subgroup analysis based on other important parameters such as the location of tumor and endoscopic techniques were also impracticable.

## Conclusion

The current meta-analysis demonstrated that the long-term survival of cT1N0 esophageal cancer patients undergoing ED was not worse than that of patients undergoing esophagectomy. ED may be considered as the primary treatment for cT1N0 esophageal carcinoma patients. However, more prospective high-quality studies are still needed to verify above findings.

## Data Availability

The original contributions presented in the study are included in the article/supplementary material, further inquiries can be directed to the corresponding author/s.
